# Chemical, Sensory, and Nutraceutical Profiling, and Shelf-Life Assessment of High-Quality Extra Virgin Olive Oil Produced in a Local Area near Florence (Italy)

**DOI:** 10.3390/molecules30132811

**Published:** 2025-06-30

**Authors:** Carlotta Breschi, Lorenzo Cecchi, Federico Mattagli, Bruno Zanoni, Tommaso Ugolini, Francesca Ieri, Luca Calamai, Maria Bellumori, Nadia Mulinacci, Fabio Boncinelli, Valentina Canuti, Silvio Menghini

**Affiliations:** 1 Department of Agricultural, Food, Environmental, and Forestry Sciences and Technologies (DAGRI), University of Florence, Via Donizetti 6, 50144 Firenze, Italy; carlotta.breschi@unifi.it (C.B.); federico.mattagli@unifi.it (F.M.); bruno.zanoni@unifi.it (B.Z.); tommaso.ugolini@unifi.it (T.U.); luca.calamai@unifi.it (L.C.); fabio.boncinelli@unifi.it (F.B.); valentina.canuti@unifi.it (V.C.); silvio.menghini@unifi.it (S.M.); 2 Institute of Bioscience and BioResources (IBBR), National Research Council of Italy (CNR), Via Madonna del Piano 10, Sesto Fiorentino, 50019 Florence, Italy; francesca.ieri@cnr.it; 3 Department of Neurofarba, University of Florence, Via Ugo Schiff 6, Sesto Fiorentino, 50019 Florence, Italy; maria.bellumori@unifi.it (M.B.); nadia.mulinacci@unifi.it (N.M.)

**Keywords:** geographic origin, storage, bottle size, typicity, consumer preferences, cognitive salience index, nutritional value score, LOX-pathway, volatile compounds, phenolic compounds

## Abstract

Consumers are increasingly willing to pay a premium for high-quality extra virgin olive oils (HQ-EVOOs) with specific sensory or nutraceutical properties, and originating from particular botanical or geographical origins. Regarding geographic origin, Italy is one of the main producers, with many local production areas, each characterized by its own distinctive typicity. The aim of this study is the chemical, sensory, and nutraceutical profiling of HQ-EVOO produced over two production years in Montespertoli (province of Florence) by 12 producers involved in the “MontEspertOlio” project, funded by the Tuscan Region. Oils were produced based on a production process previously defined and specifically applied to this territory. The shelf-life of the oil was also evaluated over a 12-month period. Legal quality parameters were analyzed according to EU regulation. Phenolic compounds, tocopherols, fatty acid composition, and volatile compounds were analyzed using HPLC-DAD, HPLC-FLD, HS-SPME-GC-MS, and GC-FID, respectively. Finally, sensory analysis was conducted using the Panel Test method. Results showed that Montespertoli HQ-EVOO is characterized by distinctive sensory and chemical traits that fully match consumer preferences, even across two production years characterized by different growing conditions. The shelf-life performance was excellent over 12 months, also showing a protective effect of greater bottle sizes against oxidation.

## 1. Introduction

Virgin olive oils (VOOx) are oils obtained from the fruit of the olive tree (*Olea europaea* L.) solely by mechanical or other physical means under conditions that do not lead to alterations in the oil [1]. Based on a series of chemical parameters and the presence or absence (and intensity) of certain sensory attributes (e.g., positive fruity notes and sensory defects), VOOx are commercially classified into extra virgin olive oil (EVOO), virgin olive oil (VOO), or lampante virgin olive oil (LVOO) [2,3]. Among these, EVOO represents the highest quality category, encompassing all VOOx that are free from sensory defects and possess a perceptible fruity note. EVOO includes both oils from various botanical and geographical origins, which differ in intensity of sensory attributes (e.g., fruitiness, bitterness, pungency), and oils with distinct sensory and nutraceutical profiles. These variations depend, for instance, on the degree of olive ripeness, cultivar, and geographical origin. Thus, assessing the overall quality of an oil requires considering not only its commercial classification, but also its sensory and nutritional properties. Given the increasing consumer demand for high-quality, traceable, and health-promoting EVOOs, as well as the need for more precise valorization of local productions, advancing knowledge on the chemical, sensory, and nutraceutical attributes of olive oils from specific territories is of both scientific and socio-economic relevance.

Breakthroughs in current global research on virgin olive oil quality include advanced analytical techniques for authentication and quality control [4,5], studying the influence of cultivar, terroir, and agronomic practices on oil quality [6,7], evaluating health-related functional compounds [8,9,10], innovations in shelf-life and packaging [11], and regulatory and standardization advances.

The nutritional benefits of EVOO are well documented in the literature [12,13,14]. Based on scientific evidence, the European Food Safety Authority (EFSA) has approved health claims related to EVOO [15,16], including claims regarding tocopherols and unsaturated fatty acids (also found in other foods), as well as a specific health claim for phenolic compounds in virgin olive oil [17]. Nevertheless, these claims are still rarely used by EVOO producers and bottlers, mainly because they are considered insufficiently clear or understandable to consumers [14]. To address this, a Nutritional Value Score (*NVS*) has been recently proposed, using an algorithm to assess the nutritional value of EVOO on a scale from 1 to 100, based on chemical analysis data [10].

The sensory and nutritional quality of EVOO primarily depends on several compounds not currently considered by the EU Regulation for commercial classification. These include phenolic compounds (PCs), volatile organic compounds (VOCs), and tocopherols. PCs are primarily composed of secoiridoid derivatives, formed after the de-glycosylation of oleuropein and ligstroside by β-glucosidase during olive milling. Other PCs include lignans and small amounts of flavonoids, phenolic acids, and phenolic alcohols [18,19,20,21]. VOCs represent a highly complex group of hundreds of compounds, including alcohols, esters/lactones, carboxylic acids, aldehydes, ketones, hydrocarbons, terpenes, and furans. Their concentration varies and significantly contributes to both positive (primarily via lipoxygenase pathway (LOX) products and terpenes) or negative sensory attributes in VOO [5,22,23,24,25,26,27]. Tocopherols (Vitamin E) exist in four isoforms (i.e., α, β, γ, δ), with α-tocopherol being the predominant form in VOO [28,29]. The profile of these compounds, and thus the sensory and nutritional quality of EVOO, is influenced by numerous factors, including botanical and geographical origin. Consequently, an increasing number of consumers are willing to pay a premium for oils with a recognized origin [30,31,32,33,34,35,36]. Accordingly, characterizing EVOOs from specific territories contributes to their valorization, making them more recognizable and appealing to consumers. However, this holds true only when the oil, marketed as originating from a given territory, truly exhibits the typical characteristics of oils from that region.

A relevant example is the case of numerous small producers in the Montespertoli area (Province of Florence, Tuscany, Italy), who have been producing EVOO for many years, often using specialized nearby olive mills. The “MontEspertOlio” project was initiated with the aim of enhancing the competitiveness of the local olive oil sector. To this end, four strategic actions were identified: (i) producing EVOOs of the highest possible quality by adapting well-established production protocols to the local context; (ii) conducting a thorough sensory, chemical, and nutraceutical profiling of the EVOO produced, both at production and over storage; (iii) evaluating whether the oils’ profile align with consumer preferences, and (iv) developing territorial branding initiatives, to increase the recognizability and link it to its place of origin in the eyes of consumers.

Regarding point (i), it is important to note that EVOO quality is influenced by numerous factors before, during, and after oil extraction. Pre-extraction factors include cultivar, geographical origin, agronomic and pedoclimatic conditions, damage to olives from pests or weather, ripening stage, and harvesting and storage methods. Processing-related factors include both horizontal (e.g., mill hygiene and process scheduling) and vertical aspects (e.g., post-harvest fruit handling and the extraction steps: crushing, malaxation, and oil separation via decanter centrifugation). Post-harvest includes storage methods, with emphasis on stabilization by filtration, bottling practices, marketing logistics, protection from light, heat, and oxygen exposure [37,38,39].

To implement these actions and achieve their associated goals, the Montespertolio project received funding from the Tuscany Region (Italy). The project partnership includes, among others, two olive mills and twelve small VOO producers, all of whom have expressed strong interest in improving both the quality and marketability of their EVOO. This collective effort enables the sharing of production know-how and commercial strategies, providing both individual and collective benefits to the participants.

This article focuses on objectives (i) to (iii) outlined above. Specifically, the study aims to (i) characterize EVOOs from Montespertoli in terms of their chemical, sensory, and nutraceutical profiles; (ii) monitor the evolution of these characteristics during one year of storage for two selected oils stored in two different bottle sizes; (iii) evaluate the relevance of chemical, sensory and nutraceutical characterization attributes in influencing consumer preferences. To this end, 12 EVOOs were produced by 12 small farms over two consecutive years, and subjected to comprehensive analysis. Two of these oils were selected for a shelf-life study conducted under ideal storage conditions (i.e., no light or oxygen exposure, room temperature) using two different bottle sizes. Finally, a market survey was conducted involving 700 regular Italian EVOO consumers.

## 2. Results and Discussion

### 2.1. EVOO Production

The EVOOs produced within the MontEspertOlio project were obtained following up-to-date processing guidelines specifically developed and optimized for the transformation of Tuscan olives. These protocols take into account the local characteristics of olive oil production, which primarily involve local cultivars and olives from the Tuscan territory. The applied protocols were developed in the context of earlier projects funded by the Tuscany Region under the 2007–2013 Rural Development Programme (PSR), such as (i) “Protocolli innovativi per la produzione di olio extravergine nella realtà aziendale Toscana”; (ii) the project OLEOSALUSISTEM; (iii) the project OLEOTEKINNOVA. They aim to produce high-quality EVOO with enhanced sensory and nutritional properties by employing technologies designed to minimize both environmental and oxidative degradation. The ultimate goal is to maximize the extraction of key quality-enhancing compounds such as phenolic compounds, volatile compounds, and tocopherols (vitamin E). The key steps in the production process, conducted over two years (i.e., 2023 and 2024) are detailed in Section 3.2, and briefly include (i) harvesting healthy olives and milling within 24 h; (ii) washing, debranching and leaf removal; (iii) crushing using of a metal knife crusher; (iv) malaxation with a closed, vertical-axis malaxer; (v) separation via a two-phase decanter without water addition; (vi) immediate filtration using cellulose fiber filter sheets to remove turbidity, without prior storage.

For characterizing and studying the shelf-life of the produced EVOOs, both chemical and sensory analyses were performed. In addition to the standard analyses required for the commercial classification of virgin olive oils, the study also included components responsible for sensory and nutraceutical quality, such as VOCs, phenolic compounds, and tocopherols. Furthermore, the Nutritional Value Score (NVS) was used to objectively assess the nutritional quality of the oils [10].

### 2.2. Profiling of Montespertoli EVOO

#### 2.2.1. Chemical, Sensory, and Nutritional Characterization

Table 1 shows the chemical characteristics of the 12 EVOOs produced over the two production years, including free acidity, peroxide value, spectrophotometric indices, total tocopherols, and total phenolic compounds. Table 2, Table 3 and Table 4 provide data on volatile compounds, sensory attributes, and Nutritional Value Score (*NVS*), respectively. Further details concerning the values of individual tocopherols, major phenolic compounds, and LOX VOCs are reported in Table S1 (phenols), Table S2 (tocopherols), and Table S3 (LOX-related VOCs) of the Supporting Information File.

All quality parameters remained well below the maximum limit established by the European Regulation [2,3] for the extra virgin category (i.e., free acidity ≤ 0.80%; peroxide value ≤ 20.0 meq_O2_/kg; K_232_ ≤ 2.50; K_270_ ≤ 0.22). Specifically, in 2023 and 2024, respectively, free acidity ranged 0.20–0.27% and 0.14–0.28%; peroxide value 4.2–8.3% and 4.8–9.6 meq_O2_/kg; K_232_ 1.58–1.80 and 1.65–1.80; K_270_ 0.12–0.15 and 0.13–0.21. Along with the absence of sensory defects and a median of fruity greater than 0 (Table 3), these values confirmed that all oils produced qualified as extra virgin. Low values of free acidity confirmed the good quality of the olives, while the low oxidation indices (peroxides, K_232_, K_270_) highlighted the effectiveness of the processing protocol in keeping the level of oxidation low. Table 1 also shows high contents of antioxidant compounds: total tocopherols ranged 263.0–467.6 mg/kg (mean, 369.3 mg/kg) in 2023 and 266.9–544.9 mg/kg (mean, 345.6 mg/kg) in 2024. Total phenolic content ranged 423.1–663.6 mg/kg (mean, 540.5 mg/kg) in 2023 and 338.8–569.7 mg/kg (mean, 447.0 mg/kg) in 2024, all values well-above the 250 mg/kg threshold for the EFSA health claim for EVOO phenolic compounds [17]. These data confirm that the adopted protocol is suitable for extracting high levels of antioxidants, thus contributing to the nutraceutical value of the oil.

Table S1 of the Supplementary Material shows low contents of hydroxytyrosol and tyrosol in both 2023 (averaging 3.2 and 4.3 mg/kg) and 2024 (averaging 1.6 and 2.7 mg/kg, respectively). Consequently, the hydrolysis ratio (defined as the sum of tyrosol and hydroxytyrosol relative to total phenolic content [39]) remained low, below 1.9% and 2.3% in 2023 and 2024, respectively, indicating a non-degraded phenolic profile. Oleacein and oleocanthal (including hydroxylated forms) were the predominant phenolic compounds, accounting for 50–90% of the total phenolic content, while some variability was observed in lignan content between years. Overall, the Montespertoli oils produced according to the protocol adopted in the project are characterized by (i) total phenolic contents ≥ 350 mg/kg; (ii) low levels of tyrosol and hydroxytyrosol; (iii) a non-degraded phenolic profile, with a low hydrolysis ratio; and (iv) high levels of oleacein and oleocanthal, (≥50% of total phenolic content).

Table S2 of the Supplementary Material shows that α-tocopherol was the predominant tocopherol form, followed by β + γ- and low levels of δ, in agreement with previous literature [28,29].

Concerning the 71 analyzed VOCs, Table 2 shows that the oils had a mean total VOC content of 65.4 mg/kg, largely represented by LOX-derived VOCs (71% and 89% in 2023 and 2024, respectively), in turn mainly constituted by (*E*)-2-hexenal (87% and 90% of LOX VOCs in 2023 and 2024, respectively): noteworthy, (*E*)-2-hexenal was in a very narrow range (83–90% and 88–91% in 2023 and 2024, respectively). On the contrary, oxidation- (i.e., “rancid VOCs”) and microbial-related (i.e., “microbial VOCs”) VOCs were present in low amounts. Detailed data of LOX-derived VOCs (Table S3A,B) show that besides (*E*)-2-hexenal, the highest contents were found for 1-penten-3-one, (*Z*)-3-hexenol, and (*E*)-2-hexenol, ranging 1–3% in most samples. All other compounds generally showed levels below 1%. Among compound groups, the highest percentages were therefore those of alcohols, followed by ketones and aldehydes other than (*E*)-2-hexenal, while ester contents were below 1%. These findings indicate that the oils of Montespertoli produced according to the protocol adopted in the project are characterized by (i) volatile profiles rich in LOX-derived VOCs and low in molecules associated with sensory defects; (ii) LOX-derived VOCs represented by (*E*)-2-hexenal ≥ 80%; (iii) ester contents < 1% of LOX VOCs; and (iv) LOX profiles characterized by the presence of 1–3% of 1-penten-3-one, (*Z*)-3-hexenol, and (*E*)-2-hexenol.

Table 3 shows medium intensity for the main sensory attributes. With very few exceptions, oils’ sensory profiles were consistent across the two production years and producers, with almond and artichoke being dominant notes. No sensory defects were detected, aligning with VOC profile.

Table 4 presents the nutritional value measured in the oils using the NVS algorithm, taking into account phenolic compounds, tocopherols, and fatty acid composition [10]. All oils scored above 80 in 2023 and above 85 (except one sample) in 2024, confirming the efficient combination of olive quality and processing protocol in enabling the extraction of health-promoting compounds.

#### 2.2.2. Commercial Category of the Montespertoli Oils and Their Profile

The first important finding emerging from the reported data is that all the oils produced were classified as extra virgin, fulfilling the primary requirement for offering a product that, even before being clearly recognizable to consumers for its origin-related characteristics, can be appreciated as belonging to the highest commercial category.

With rare exceptions, all oils shared a consistent profile characteristic of the Montespertoli oils produced with the processing protocol employed:

- Free acidity ≤ 0.30%
- Peroxide value ≤ 10.0 meq_O2_/kg
- K_232_ ≤ 1.90
- K_270_ ≤ 0.19
- Total phenolic content ≥ 350.0 mg/kg
- Hydrolysis ratio ≤ 2.0% at production
- Oleacein + oleocanthal content ≥ 50% of the total phenolic content
- Total tocopherol content ≥ 250.0 mg/kg
- Total LOX VOC content ≥ 30.0 mg/kg
- (*E*)-2-hexenal ≥ 80% of total LOX VOCs
- LOX esters ≤ 1% of total LOX VOCs
- 1-penten-3-one, (*Z*)-3-hexenol, and (*E*)-2-hexenol: 1–3% of LOX VOCs
- Median of fruitiness ≥ 3
- Median of bitterness ≥ 3
- Median of pungency ≥ 4
- Fruity profile characterized by a prevalence of almond and artichoke.
- NVS ≥ 80.0

#### 2.2.3. The Profile of EVOO and the Consumer Preferences

To assess whether the defined EVOO profile aligns with current consumer preferences, chemical, sensory, and nutritional data were compared with results from a market online survey of January 2025 involving 700 regular Italian EVOO consumers recruited by a marketing research firm. Through this survey, relevant attributes in consumer preferences were examined in terms of cognitive salience using a specific *Cognitive Salience Index* (*CSI*).

Seven main semantic categories emerged (Table 5), each with its own degree of consistency; even the last one, although minimally represented, still holds interpretative significance.

By comparing the chemical and sensory data with the CSI, a strong alignment occurred between objective analytical values and subjective perceptions, at the level of three semantic categories. The semantic category “Taste and Flavor” was the most salient (CSI = 0.5366; frequency = 527 mentions), including descriptors like flavor, delicate, intense, spicy, bitter, and full-bodied. These descriptors aligned closely with sensory outcomes (bitterness, pungency, aromatic intensity) and are directly supported by the high phenolic and LOX VOC content, known to elicit such retronasal sensations. Likewise, the category “Aroma and Scent” (CSI = 0.1795; frequency = 198) reflected the volatile profile, especially the presence of green aldehydes like (*E*)-2-hexenal. The consumers’ associations are in clear agreement with the analytical data. Similarly, the cognitive salience of “Health and Wellness” (CSI = 0.1316) aligns with the consistent presence of phenolic compounds and tocopherols, recognized for their antioxidant and protective roles. This alignment is further reinforced by the high Nutritional Value Score (NVS ≥ 80 and ≥ 85 in 2023 and 2024, respectively) of the examined EVOO, which confirms its strong nutritional profile and supports the perception of the oil as a health-promoting and functional food.

In addition to these categories, the semantic category related to the “visual appearance” of the product is relevant, with consumers associating an EVOO with certain colors and transparency. Significant, and directly related to the semantic category “Health and Wellness,” is the category “Naturalness and authenticity”, in which product quality is linked to the terms natural, organic, genuine, and authentic. Less pronounced but still significant is the attention to the origin and typicality, especially if Italian. Finally, the last semantic category, “Price and value”, indicates a sensitivity to price that is sometimes amply justified but other times considered too high.

### 2.3. Shelf-Life Study

Like all fatty products, EVOO is prone to oxidation during storage, leading to rancidity [40]. Furthermore, in unfiltered oils, the simultaneous presence of microorganisms/enzymes, water, and organic matter can cause microbial degradation phenomena, resulting in a sensory defect known as “fusty/muddy sediment” [41]. These defects compromise the sensory quality of the oil, leading to commercial category downgrading and reducing consumer appreciation [2,3].

To minimize microbiological degradation, immediate filtration is a proven and widely recommended strategy [41], and therefore, it was applied to all oils produced within the MontEspertOlio project. For oxidation, reducing exposure to light, temperatures, oxygen, pigments, and metal ions significantly slows the processes, though it does not completely prevent it [42]. Thus, it is still necessary to develop storage strategies that further reduce the impact of oxidation on oil quality.

This shelf-life study investigated the impact of bottle size on the quality of two EVOOs during 12-month storage (Section 3.3). The hypothesis, supported by analogous observations in wine [43,44], was that larger bottles provide better protection against oxidation, due to (i) smaller surface-to-volume ratio, reducing light exposure; (ii) the same oxygen amount acting on a larger oil mass; (iii) in cases of sudden temperature changes, slower heat transfer across full oil mass; (iv) lower metal ion concentration leached from container surfaces.

#### 2.3.1. Evolution of Chemical Characteristics over Time

**Figure 1:**
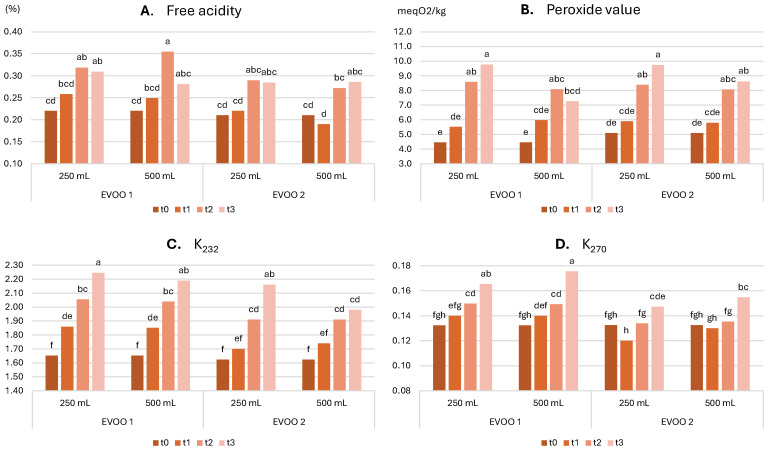
Evolution of chemical quality parameters (**A**) free acidity, (**B**) peroxide value, (**C**) K_232_, and (**D**) K_270_ in the two oils stored in the two bottles over storage. t0, time 0; t1, 2 months; t2, 5 months; t3, 12 months; 250 mL and 500 mL are the bottles’ sizes. In each graph, different letters over the bars indicate significant differences at *p* < 0.05.

**Legal quality parameters**: Figure 1A–D shows the evolution of key chemical quality parameters (i.e., free acidity, peroxide value, spectrophotometric indices) over time, while Table 6 summarizes the statistical significance of the effect of each factor and their interactions, as evaluated using 3-way ANOVA. Detailed data are also provided in Table S4 of the Supplementary Material.

Free Acidity: Table 6 shows that free acidity varied significantly with oil type, storage time, and their interaction. No significant effects were observed in relation to bottle size or its interaction with other factors. Free acidity increased from an initial value of 0.22% to 0.31% in the 250 mL bottle and to 0.28% in the 500 mL bottle for EVOO 1, and from an initial value of 0.21% to 0.28% in the 250 mL bottle and to 0.29% in the 500 mL bottle for EVOO 2. These values are considered acceptable for high-quality oils after one year of storage. Across various combinations of oil type and bottle size, a general trend of increasing free acidity values was observed. This increase is due to the spontaneous, though slow, hydrolysis of triglycerides, resulting in the formation of free fatty acids and partial glycerides [38]. As expected, bottle size had no significant effect on this parameter, as hydrolysis is not influenced by oil mass or surface-to-volume ratio.

Peroxide Value: Table 6 indicates that peroxide value was significantly affected by storage time, bottle size, and their interaction. Peroxide values increased from an initial 4.5 meq_O2_/kg_oil_ to 9.8 meq_O2_/kg_oil_ in the 250 mL bottle and to 7.3 meq_O2_/kg_oil_ in the 500 mL bottle for EVOO 1, and from an initial 5.1 meq_O2_/kg_oil_ to 9.7 meq_O2_/kg_oil_ in the 250 mL bottle and to 8.6 meq_O2_/kg_oil_ in the 500 mL bottle for EVOO 2. These values are still acceptable for high-quality oils after one year of storage. The increase in peroxide value during oil storage is well-documented, often showing an initial rise, and a subsequent decline in sealed containers with limited oxygen availability [45,46,47]. For both EVOOs, Figure 1B shows a continuous and relatively steady increase in the 250 mL bottles, while a rise followed by a plateau is observed for the 500 mL bottles. This suggests that the greater oil mass in the larger bottles consumes oxygen more quickly, thereby slowing peroxide formation. These findings confirm the better performance of larger bottles in slowing oxidation processes.

K_232_: Table 6 shows that K_232_ values were significantly influenced by storage time, EVOO sample, and their interaction, as well as the interaction between time and bottle size. The latter significance indicates that K_232_ increased over time to varying extents depending on bottle size. K_232_ values ranged from an initial value of 1.65 to 2.25 in the 250 mL bottle and to 2.19 in the 500 mL bottle for EVOO 1, and from 1.62 to 2.16 in the 250 mL bottle and to 1.98 in the 500 mL bottle for EVOO 2. These values are still within acceptable limits for high-quality oils. A consistent increase was observed under all conditions (Figure 1C), more pronounced in the 250 mL bottles. The rise in K_232_ is primarily associated with the formation of conjugated dienes in the early stages of lipid oxidation. These results further confirm that larger bottles more effectively limit oxidation. In comparison with previous research that demonstrated improved protection in larger bottles under light exposure [48], these findings suggest that even in the dark, larger bottles better preserve EVOO’s quality from oxidation.

K_270_: Table 6 indicates that the K_270_ was significantly influenced by storage time, oil sample, and bottle size. Additionally, significant effects for the interaction between oil sample and storage time, as well as between storage time and bottle size, were observed. K_270_ values ranged from an initial value of 0.13 to 0.17 in the 250 mL bottle and to 0.18 in the 500 mL bottle for EVOO 1, and from 0.13 to 0.15 in both bottle sizes for EVOO 2. These values are also considered acceptable for high-quality oils after one year of storage. A consistent increase in K_270_ was observed for EVOO 1, while in EVOO 2, the increase occurred primarily in the final months of storage (Figure 1D). The increase in K_270_ is associated with the formation of conjugated trienes and secondary oxidation products such as dialdehydes. Therefore, unlike peroxide value and K_232_, the larger bottle did not exhibit a clearly beneficial effect on K_270_.

**Figure 2:**
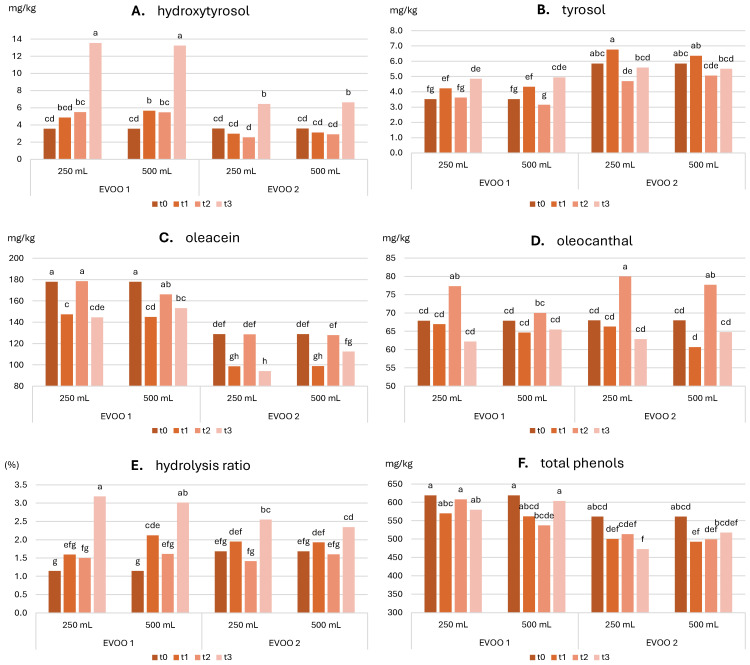
Evolution of phenolic compounds (**A**) hydroxytyrosol, (**B**) tyrosol, (**C**) oleacein, (**D**) oleocanthal, (**E**) hydrolysis ratio, (**F**) total phenolic content) in the two oils stored in the two bottles over storage. t0, time 0; t1, 2 months; t2, 5 months; t3, 12 months; 250 mL and 500 mL are the bottles’ sizes. In each graph, different letters over the bars indicate significant differences at *p* < 0.05.

**Phenolic compounds**: Figure 2 shows the evolution of the total phenolic content (TPC), the main individual phenols (i.e., hydroxytyrosol, tyrosol, oleacein, oleocanthal), and the hydrolysis ratio (i.e., the ratio of the sum of tyrosol and hydroxytyrosol divided by TPC) over time, while Table 7 summarizes the significance of the effect of each factor and their interaction, as evaluated using a 3-way ANOVA. Additional data are provided in Table S5 of the Supplementary Material.

Phenolic compounds are among the most important molecules characterizing high-quality extra virgin olive oils (EVOOs) and, along with VOCs, represent a key parameter for distinguishing the quality of different EVOOs. These compounds confer multiple attributes to EVOO, including antioxidant properties that enhance shelf-life, sensory properties (contributing to the oil’s typical bitter and pungent characteristics), and nutritional properties that help reduce the risk of various degenerative diseases [21,49]. The most distinctive EVOO phenols are secoiridoid derivatives of oleuropein and ligstroside. This report focuses on phenolic molecules relevant for profiling of Montespertoli’s EVOOs: (i) total phenolic content (TPC) that provides a general indication of how rich the EVOO is in phenolic compounds; (ii) oleacein and oleocanthal, often the most abundant phenols in high-quality EVOO, though their content vary depending on cultivar; (iii) tyrosol and hydroxytyrosol, as final degradation products of the EVOO’s phenolic compounds resulting from hydrolytic, esterase, and intramolecular rearrangement processes. In freshly produced high-quality EVOO, their concentration is low and negligible relative to the TPC, but they increase over time, and high levels indicate a degraded phenolic profile; (iv) hydrolysis ratio, defined as the ratio between the sum of tyrosol and hydroxytyrosol to the total phenolic content. The higher this value, the more advanced the hydrolytic degradation of the phenolic profile, indicating a greater degree of EVOO aging [39,50,51].

Tyrosol and Hydroxytyrosol: Table 7 shows that the concentrations of these two molecules varied significantly with storage time, sample type, and their interaction. No significant effects were observed due to bottle size. Hydroxytyrosol values ranged from an initial 3.6 mg/kg to 13.5 mg/kg for the 250 mL bottle and 13.2 mg/kg for the 500 mL bottle in EVOO 1, and from 3.6 mg/kg to 6.4 mg/kg (250 mL) and 6.6 mg/kg (500 mL) in EVOO 2. Tyrosol values ranged from an initial 3.5 mg/kg to 4.8 mg/kg (250 mL) and 4.9 mg/kg (500 mL) in EVOO 1, and from 5.9 mg/kg to 5.6 mg/kg (250 mL) and 5.5 mg/kg (500 mL) in EVOO 2. These values are also acceptable for high-quality oils after one year; only in the case of hydroxytyrosol in EVOO 1, a significant increase occurred at t3, suggesting the beginning of a significant degradation of oleuropein derivatives. Tyrosol content did not follow a consistent trend over time, while hydroxytyrosol showed a marked increase at the final storage time, following an unsystematic pattern during the early months (Figure 2A,B). This increase was especially notable in EVOO 1.

Oleacein and Oleocanthal: Table 7 shows that the levels of both compounds varied significantly with storage time and the interaction between storage time and bottle size. Oleacein content also varied significantly with oil sample, and oleocanthal with interaction between sample and storage time. Oleacein values ranged from an initial 177.9 mg/kg to 144.5 mg/kg (250 mL) and 153.2 mg/kg (500 mL) in EVOO 1, and from 128.8 mg/kg to 94 mg/kg (250 mL) and 112.6 mg/kg (500 mL) in EVOO 2. These are considered good values for high-quality oils after one year. Oleocanthal levels ranged from an initial 67.9 mg/kg to 62.2 mg/kg (250 mL) and 65.5 mg/kg (500 mL) in EVOO 1, and from 68 mg/kg to 62.8 mg/kg (250 mL) and 64.8 mg/kg (500 mL) in EVOO 2. These values are also acceptable for high-quality EVOOs after one year of storage. Figure 2C shows higher oleacein levels in EVOO 1, with a general decreasing trend over time, more pronounced in the smaller bottle, despite some irregularities likely due to an outlier at T2. This trend, not observed for oleocanthal, can be attributed to the molecular structures: oleacein contains an *o*-diphenolic ring more susceptible to oxidation than the monophenolic ring of oleocanthal. This explains the time-dependent decline in oleacein and the more stable levels of oleocanthal, as well as the better preservation effect of the larger bottle [52].

Total Phenolic Content: Table 7 shows that TPC varied significantly with storage time, sample type, and the interaction between storage time and bottle size. A significant decrease was observed only in EVOO 2 stored in 250 mL bottles, supporting the superior performance of larger bottles. TPC ranged from an initial 618.8 mg/kg to 579.9 mg/kg (250 mL) and 603.9 mg/kg (500 mL) in EVOO 1, and from 560.9 mg/kg to 472.4 mg/kg (250 mL) and 517.4 mg/kg (500 mL) in EVOO 2. These values are still very good for high-quality EVOOs after one year of storage.

Hydrolysis Ratio: Table 7 shows that the hydrolysis ratio varied significantly with storage time and with its interaction with both sample and bottle size. Hydrolysis ratios ranged between 1 and 3% for both oils. Figure 2E indicates that, after an initial period with no consistent trend across treatments, a marked increase in the hydrolysis ratio occurred at the final storage point. This increase was particularly pronounced in EVOO 1, with a slight predominance in the 250 mL bottle for both oils.

**Figure 3:**
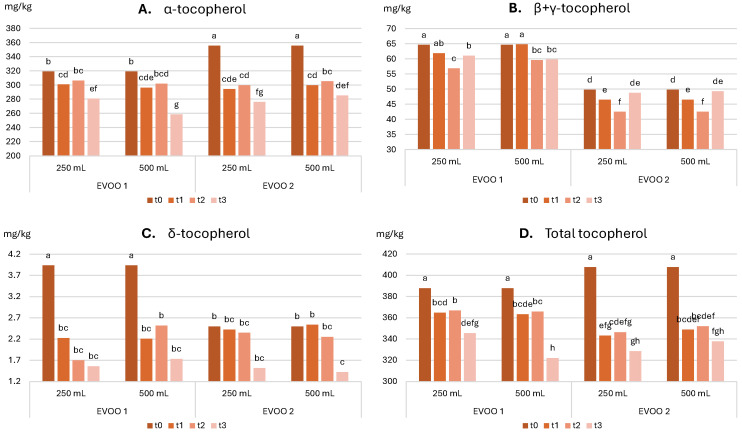
Evolution of tocopherols (**A**) α-, (**B**) β + γ, (**C**) δ, (**D**) Total content in the two oils stored in the two bottles over storage. t0, time 0; t1, 2 months; t2, 5 months; t3, 12 months; 250 mL and 500 mL are the bottles’ sizes. In each graph, different letters over the bars indicate significant differences at *p* < 0.05.

**Tocopherols**: Figure 3 shows the evolution of total tocopherol content (TTC), and of individual forms (i.e., α, β + γ, δ) over time. Table 8 summarizes the significance of the effect of each factor and of their combination as evaluated using 3-way ANOVA. Detailed data are also provided in Table S6 of the Supplementary Material.

Tocopherols are lipophilic antioxidant molecules present in oils as α, β, γ, and δ isoforms [8], with the α form being predominant in EVOO [53]. In this study, β- and γ-forms are reported together, as they are not separated chromatographically using the method applied. Table 8 shows that TTC varied significantly with storage time, EVOO sample, and their interaction, as well as the interaction between sample and bottle size and the three-way interaction. TTC ranged from an initial value of 387.7 mg/kg to 345.5 mg/kg for the 250 mL bottle and to 322.0 mg/kg for the 500 mL bottle in the case of EVOO 1, and from an initial 407.8 mg/kg to 328.5 mg/kg for the 250 mL bottle and to 337.8 mg/kg for the 500 mL bottle for EVOO 2. These values are considered good for high-quality EVOOs after one year of storage. As shown in Figure 3, initial tocopherol content was higher in EVOO 2 but decreased markedly after t1, falling below the levels observed in EVOO 1. Both oils showed a general decreasing trend, with opposite effects from bottle size.

**Figure 4:**
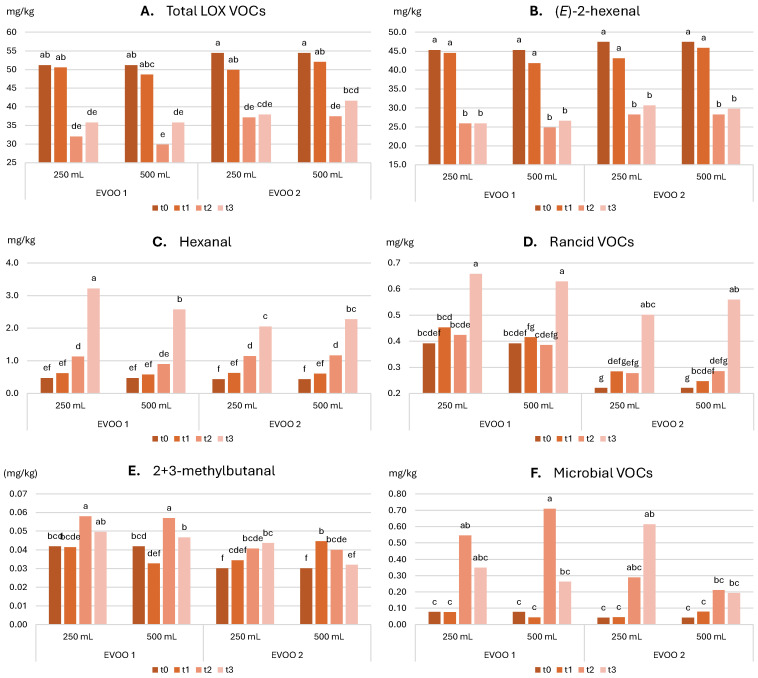
Evolution of volatile compounds. (**A**) Total LOX VOCs, (**B**) (*E*)-2-hexenal, (**C**) hexanal, (**D**) rancid VOCs, (**E**) 2 + 3 methyl butanal, (**F**) microbial VOCsin the two oils stored in the two bottles over storage. t0, time 0; t1, 2 months; t2, 5 months; t3, 12 months; 250 mL and 500 mL are the bottles’ sizes. In each graph, different letters over the bars indicate significant differences at *p* < 0.05.

**Volatile compounds**: Figure 4 shows the evolution of selected VOCs such as hexenal, (*E*)-2-hexenal, total LOX VOCs, rancid VOCs, microbial VOCs, and 2 + 3-methylbutanal over time. Table 9 summarizes the significance of each factor and their combination as evaluated using 3-way ANOVA. Detailed data are provided in Table S7 of the Supplementary Material.

VOCs are key markers in differentiating oils from a sensory perspective [5,31,36]. Major VOCs groups in EVOO include the following: (i) LOX-pathway-derived molecules, linked to green and fruity notes; (ii) oxidation-related molecules (e.g., octane, saturated C5–C10 aldehydes, mono- and di-unsaturated C7–C10 aldehydes); (iii) molecules linked to microbiological defects (with a wide spectrum resulting from numerous microbial processes) [27,31,54,55].

Hexanal and (*E*)-2-hexenal: According to Table 9, hexanal, which can be formed via both the LOX-pathway and oxidation, showed significant variation with all factors except the time–bottle size interaction. Conversely, (*E*)-2-hexenal varied mainly with time and, to a lesser extent, with the sample. Table 9 shows a steady increase in hexanal across all conditions, more pronounced in the 250 mL bottle of EVOO 1 and similarly rising in EVOO 2. This suggests that larger bottles slow down secondary oxidative processes. (*E*)-2-Hexenal, the most abundant VOC in all EVOOs over the whole storage period, showed a sharp decrease starting from t2, with few significant changes thereafter.

LOX VOCs: LOX VOC content varied significantly with storage time and sample type. The trend observed in Figure 4A reflects the patterns seen for (*E*)-2-hexenal (dominant and decreasing) and hexanal (increasing), with a strong decrease at t2 driven by the former and a slight increase at t3, particularly in EVOO 1, driven by the latter.

Rancid VOCs: Table 9 shows that the content of molecules linked to oxidation varied significantly with storage time, sample, sample–time and sample–bottle interactions, and the three-way interaction of time–sample–bottle size. Figure 4D shows an overall increase, especially in the final storage stage. Bottle size had no significant protective effect, aligning with what was observed for K_270_.

Microbial VOCs and 2 + 3-methylbutanal: According to Table 9, microbial VOCs varied significantly with storage time and their interaction with the sample. Figure 4F shows a marked increase from t1 to t2 in EVOO 1, followed by a decrease at t3. For EVOO 2, there was a consistent increase in the 250 mL bottle and a milder or absent increase at t3 in the 500 mL bottle. No clear trend was observed for 2 + 3-methylbutanal (molecules related to the development of the “fusty” defect [54] (Figure 4E). This confirms that, under the present conditions (filtered oil), the increase in these molecules is not significant.

#### 2.3.2. Evolution of Sensory and Nutritional Characteristics over Time

**Sensory parameters**: Table 10 shows the evolution of key sensory characteristics during storage. Both EVOOs remained free from sensory defects throughout the storage period (Table 10). Tastings were conducted using freshly opened bottles stored under identical conditions. Regarding the fruity attribute, panelists found ripe rather than green fruitiness in both oils in 2023, likely due to the excessive heat at harvest. The absence of defects confirms the quality of processing. In EVOO 1, the fruity note started at a score of 4 and remained stable, with a slight increase for both bottle sizes. In EVOO 2, the initial score was about 3.8, and then, it increased to 5.9 and 6.5 in the 250 mL and 500 mL bottles, respectively, followed by a decreasing trend in both bottles. Bitterness remained more pronounced during the whole storage period than at the beginning. Pungency increased from t0 to t1, and slightly decreased thereafter. This increase observed after the first weeks of storage was possibly due to sensory stabilization typical in fresh oils. This was also supported by the detailed positive VOCs data, harmony, complexity, and persistence. Overall preference differences between bottle sizes were minimal. Regarding specific descriptors, the oils were characterized by almond, artichoke, and walnut notes.

**Figure 5:**
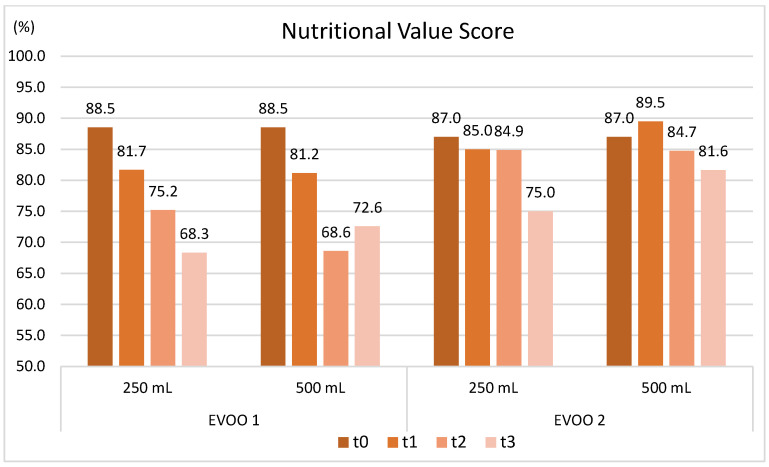
Evolution of the Nutritional Value Score (NVS) in the two oils stored in the two bottles over storage. t0, time 0; t1, 2 months; t2, 5 months; t3, 12 months; 250 mL and 500 mL are the bottles’ sizes.

Nutritional Characteristics: Figure 5 shows the evolution of the Nutritional Value Score (NVS) over time.

The nutritional value of the EVOOs was assessed using the NVS parameter, which converts chemical data into a score from 1 to 100 easily interpretable by consumers [10]. Figure 5 shows high initial NVS, scoring 87.0 for EVOO 2 and 88.5 for EVOO 1. EVOO1 also exhibited a steady decline over time, with better preservation in the 500 mL bottle. EVOO 2 showed a slower, less marked decline, again with the 500 mL bottle better preserving nutritional value.

## 3. Materials and Methods

### 3.1. Chemicals and Reagents

Ultrapure water (Milli-Q system, Millipore, Molsheim, France) was used for extractions and high-performance liquid chromatography (HPLC) analyses. HPLC-grade acetonitrile (Panreac, Spain), methanol, and orthophosphoric acid (>85%) (Merck, Darmstadt, Germany) were used for chromatographic analysis. Extraction solvents included analytical-grade methanol and ethanol (Merck, Darmstadt, Germany).

Standards used for analysis of phenolic compounds in olive oil were tyrosol and syringic acid (Merck), with the latter serving as the internal standard. For analysis of VOCs via head-space-solid phase micro extraction-gas chromatography–mass spectrometry (HS-SPME-GC-MS), 8 internal and 72 external standards (Merck Darmstadt, Germany) were used. Refined olive oil free of interfering VOCs was used for preparing standard solutions, following the MISN approach [56]. Standard of α-tocopherol (>97.5%) for analysis of tocopherols was purchased from Merck (Darmstadt, Germany).

### 3.2. Virgin Olive Oil Samples Production

Virgin olive oil samples were produced over two consecutive olive oil production years: 2023 and 2024. Twelve samples were produced each year by 12 small producers, all located in the municipality of Montespertoli (Florence, Tuscany, Italy): Tenuta Barbadoro, Azienda Agricola Fattoria di Trecento, Tenuta di Maiano, Fattorie Parri, Podere delle Falcole, Azienda Agricola Valleprima, Azienda Agricola Podere Ghiole, Azienda Agricola Solaia, Azienda Agricola le Terre di Poldo, Agriturismo Azienda Montalbino, Fattoria La Leccia, Podere Guiducci. Olives from six producers were processed by the mill of Azienda Agricola Torre Bianca (San Casciano Val di Pesa, Florence, Italy), whereas the remaining six were processed by the mill of Società Agricola Maggi (Barberino Tavarnelle, Florence, Italy). Both mills followed the same production protocol, briefly described as follows.

Olives from the typical Tuscan cultivars were harvested on the same day they were milled. In each mill, approx. 300 kg of olive fruits were processed using a plant (Mori TEM, Tavarnelle Val di Pesa, Florence, Italy) consisting of the following: (i) a cleaning line consisting of a debrancher to remove twigs, stones, and other solid debris, followed by a defoliator, a washing unit, and a drying system; (ii) a knife mill for crushing; (iii) a sealed vertical malaxer designed to reduce air–paste surface and presence of oxygen, operating at temperature below 27 °C and for < 30 min; (iv) a two-phase decanter operating without added water; (v) a filtration system including a filter using 19 cellulose cardboards in a mill, and a vertical centrifuge as a separator followed by 7 cellulose cardboards in the second mill. Processing parameters were carefully selected to minimize chemical alterations in the oils. The oils obtained were stored in 0.5-L glass bottles provided by Fara Srl Vetrerie E Cristallerie (Montespertoli, Italy), completely filled and kept in the dark at room temperature until analysis.

Minor differences were applied between the two production years. In 2023, olives were harvested over a two-week period, whereas in 2024, all olives were harvested within one week to minimize variability due to differences in the olives’ ripening degree. Furthermore, in 2024, a reduced number of cultivars was used, excluding Coratina and including at least 90% Moraiolo, Frantoio, and Leccino olives, to minimize cultivar-related variability in oils.

### 3.3. The Shelf-Life Experimental Plan

The shelf-life study was carried out, including three factors of potential variability:

1. EVOO samples: Two samples from 2023 were selected and labelled as EVOO1 and EVOO2.
2. Storage time: The oils were analyzed at four different storage times over one year:
  ⮚ Time 0 (t0): Immediately after production.
  ⮚ Time 1 (t1): After 2 months of storage.
  ⮚ Time 2 (t2): After 5 months of storage.
  ⮚ Time 3 (t3): After 12 months of storage.

These time points (2 months, 5 months, and 12 months) were chosen instead of regular intervals (e.g., 4, 8, 12 months) to better capture early-stage changes in oil quality.

3. Bottle size: Two bottle sizes were selected: 250 mL (B250) and 500 mL (B500), both provided by Fara Srl Vetrerie E Cristallerie (Montespertoli, Italy). This choice was based on the hypothesis that larger bottles better preserve oil quality, similarly to what happens in other beverages like wine [43,44].

All samples were stored under ideal conditions: no light exposure, no exposure to oxygen other than the bottle headspace, room temperature (i.e., <25 °C over the whole storage period). Each type of sample was stored and analyzed in triplicate for a total of 48 samples (2 EVOOs × 2 bottle sizes × 4 storage times × 3 replicates). The following chemical parameters were analyzed: free acidity, peroxide value, spectrophotometric indices, fatty acid composition, tocopherols, phenolic compounds, and VOCs.

### 3.4. Chemical Analysis

#### 3.4.1. Legal Quality Parameters

Free acidity (FA), peroxide value (PV), UV spectrophotometric indices (K_232_, K_270_, ∆K), and fatty acid composition were analyzed following official procedures [3].

#### 3.4.2. Analysis of Phenolic Compounds via HPLC-DAD

The analysis of phenolic compounds was carried out in accordance with the official International Olive Council method [57]. Briefly, 2 g of oil were extracted with 6 mL of a MeOH:H_2_O 80:20 mixture, consisting of 5 mL of extractive solution and 1 mL of internal standard solution (syringic acid at 0.015 mg/mL in MeOH:H_2_O 80:20). The resulting phenolic extracts were analyzed with an HP 1200 liquid chromatography system with a diode array detector (DAD, Agilent Technologies, Palo Alto, CA, USA), using a LiChrospher 100 endcapped RP-18 column (5 μm, 250 mm × 4.6 mm i.d.). Injection volume, 20 μL. Solvents (H_2_O adjusted to pH 2.0 with H_3_PO_4_, MeOH, and CH_3_CN) were eluted according to the gradient specified in the method, and chromatograms were recorded at 280 nm. Quantification was performed using syringic acid as the internal standard and tyrosol as the reference for response factor calibration. Results were expressed as mg of tyrosol equivalents per kg of oil.

#### 3.4.3. Analysis of Tocopherols via HPLC-FLD

Tocopherol analysis was conducted using high-performance liquid chromatography with fluorescence detector (HPLC-FLD). The system employed was an HP1200L liquid chromatograph (Agilent Corporation, Palo Alto, CA, USA) equipped with an autosampler, an FLD, and a C18 Lichrosorb column (5 μm, 250 × 4.6 mm i.d., Interchim, Montluçon, France). Mobile phases: (A) acidified water (pH 3.2, adjusted with formic acid), and (B) acetonitrile (CH_3_CN). The elution program was as follows: 95% B at the start, maintained for 5 min, then increased to 100% B over 1 min and held for 24 min, followed by a return to 95% B over 2 min, making a total run time of 32 min. Flow rate, 0.8 mL/min. Injection volume: 20 μL of a sample solution from 80 mg of oil in 1 mL of isopropanol. Fluorescence chromatograms were recorded at excitation and emission wavelengths of 295 nm and 325 nm, respectively [58]. Quantification was performed using a calibration curve of α-tocopherol (0–8.76 μg).

#### 3.4.4. Analysis of Volatile Organic Compounds via HS-SPME-GC-MS

The analysis of 72 volatile organic compounds (VOCs) was performed using headspace-solid phase microextraction coupled with gas chromatography–mass spectrometry (HS-SPME-GC-MS) [56]. Quantification of VOCs was carried out using the MISN approach. Briefly, 4.3 g of olive oil sample and 0.1 g of an internal standard (ISTD) solution (prepared in refined olive oil free of interfering VOCs) were added into a 20 mL screw-cap vial. The concentration of ISTDs was as follows: 4-methyl-2-pentanol, 11.8 mg/kg; 6-chloro-2-hexanone, 15.9 mg/kg; 3-octanone, 11.5 mg/kg; ethyl acetate d_8_, 10.1 mg/kg; butanol d_10_, 11.4 mg/kg; 3,4-dimethylphenol, 38.0 mg/kg; trimethylacetaldehyde, 29.1 mg/kg; acetic acid d_3_, 10.2 mg/kg). VOCs were pre-concentrated by exposing a 1-cm DVB/CAR/PDMS (50/30 µm) SPME fiber (Supelco, Belfonte, PA, USA) to the vial headspace for 20 min at 45 °C. Desorption was carried out in the injection port of a 7890a GCsystem (Agilent Technologies, Santa Clara, CA, USA) at 260 °C for 2 min in splitless mode. Separation was achieved using a DB InnoWAX capillary column (50 m × 0.2 mm i.d., 0.4 µm), and detection was performed with a quadrupole 5975c-series Mass Spectrometer detector (Agilent Technologies, Palo Alto, CA, USA). The oven temperature was initially set at 40 °C, then ramped to 156 °C at 4 °C/min, and finally to 260 °C at 10 °C/min. Helium (supplied by Nippon Gases (Sesto Fiorentino, Italy), purity > 99.999%) was used as the carrier gas at a flow rate of 1.2 mL/min. The transfer line and ion source were maintained at 250 °C and 230 °C, respectively. The MS operated in scan mode (*m*/*z* 30–350 Th, 1500 Th/s) with an ionization energy of 70 eV. Each VOC was identified by comparing mass spectra and retention times with those of authentic standards. For each compound, the most appropriate internal standard was selected, and quantification was carried out using a six-point linear calibration curve (area ratio vs. amount ratio) built from the corresponding pure standard. Further methodological details can be found in [56].

### 3.5. Sensory Analysis

Sensory analysis was performed using the Panel Test method, employing an advanced profile sheet provided by the ANAPOO association (Montevarchi, Arezzo, Italy). The profile sheet includes two sections: (i) one to assess legally defined sensory attributes for VOOx classification [2,3], including defects (rancid, fusty/muddy sediment, winey/vinegary, moldy) and positive attributes (fruity, bitterness, pungency); (ii) the other to assess orthonasal and retronasal positive attributes that distinguish the unique sensory profiles of different EVOO samples.

The Panel, officially recognized by the Italian Ministry of Agricultural Policies (MIPAAF), consisted of a panel leader and 8–12 trained assessors. Evaluations were conducted immediately after bottle opening. Oils were classified as EVOO if the median defect score was 0 and the median fruity score was >0.

### 3.6. Nutraceutical Evaluation by Applying the Nutritional Value Score (NVS)

The nutritional value of oils was assessed using the Nutritional Value Score (NVS) [10]. It is derived from an algorithm that converts chemical data into a value ranging from 1 to 100.

### 3.7. Consumers’s Preferences by Cognitive Salience Index (CSI)

Cognitive salience is an indicator for measuring the perceived importance of a concept in a collective mental context. The punctual measurement of such cognitive salience can be formulated in terms of an index, the cognitive salience index (CSI), combining term frequency and order of mention of responses by participants: the concepts reported first are considered cognitively more salient.

To achieve the CSI, the “free listing” technique was adopted, asking respondents to freely list the terms they associate with a given topic [59,60,61,62]. In the market survey carried out in this study, participants were asked to express the top five adjectives they associate with when thinking of an EVOO.

Once terms were collected, synonyms and similar expressions were identified and merged within semantic categories. Subsequently, the CSI was calculated, weighing the psychological relevance attributed to each concept (represented by the different semantic categories) by the group of respondents [63].

The semantic categories were organized in a matrix, placing the different categories on the rows and individual participants on the columns. Semantic categories mentioned by less than 10% of the respondents were excluded [64]; thus, within the scope of the present research, the 7 semantic categories indicated in the previously mentioned results are identified. Within the matrix, the position in which each semantic category is mentioned is reported for each participant. With this information, CSI was calculated for each semantic category j as:

CSIj=FjN∗APj

where

*F_j_* is the number of subjects who mentioned the characteristic *j*,

*N* is the total number of subjects,

*AP_j_* is the average of the positions of the characteristic *j*.

The CSI value was normalized between 0 and 1: when a characteristic is mentioned by all subjects (i.e., Fj = N) and always in the first position (APj = 1), the CSI takes a maximum value of 1. Since this index is not affected by the length of individual lists, it allows comparison of results obtained even between different studies [63].

### 3.8. Data Analysis

Mean and standard deviation (SD) of triplicate measurements were calculated using Microsoft Excel (Microsoft 365). A three-way ANOVA tested the effect of sample, bottle size, storage time, and their interactions on each variable. A post hoc Tukey test was used to identify significant differences. All ANOVA and Tukey analyses, along with principal component analysis, were conducted in R software (version 4.3.1, The R Foundation for Statistical Computing).

## 4. Conclusions

Results of the MontEspertOlio project confirm the importance of collaboration between virgin olive oil producers, institutions, and researchers to produce high-quality EVOO with distinctive characteristics linked to the territory of origin. Sensory, chemical, and nutraceutical characteristics have been reported for the first time, pointing out similar profiles across 2 years by 12 producers, representing the typical profile of Montespertoli’s EVOO. A further novelty comes from the relevant convergence between chemical and sensory profiling with the consumers’ preferences, quantified in terms of cognitive salience: this alignment is of strategic value for both marketing positioning and consumer education to ensure maximum commercial success of the product.

The shelf-life study allowed highlighting that Montespertoli’s EVOO remained within the extra virgin olive oil category throughout the storage period, regardless of bottle size. Nutraceutical compounds remained at high levels for the whole storage period. Sensory characteristics were confirmed as of high quality, with no sensory defects, approx. medium values of fruitiness, bitterness, and pungency, a good equilibrium of harmony, complexity, and persistence, and the presence of specific attributes such as almond and artichoke notes. Bottle size had a significant effect on preventing EVOO oxidation, with the larger sizes performing better than the smaller ones, even under dark conditions.

## Figures and Tables

**Table 1:** Chemical data of the produced EVOOs in 2023 and 2024. TTC, total tocopherol content; TPC, total phenolic content.

Sample	FA (%_oleic acid_)		PV (meq_O2_/kg)		K_232_		K_270_		∆K		TTC		TPC	
	2023	2024	2023	2024	2023	2024	2023	2024	2023	2024	2023	2024	2023	2024
**EVOO1**	0.22	0.21	4.5	9.6	1.65	1.80	0.13	0.17	0.003	0.002	387.7	268.8	618.8	532.8
**EVOO2**	0.21	0.23	5.1	5.9	1.62	1.68	0.13	0.14	0.003	0.002	407.8	317.1	560.9	409.8
**EVOO3**	0.27	0.18	7.2	6.5	1.70	1.73	0.14	0.14	0.004	0.002	373.0	326.8	596.3	396.1
**EVOO4**	0.27	0.17	4.6	5.0	1.76	1.67	0.14	0.15	0.004	0.002	369.4	322.5	663.6	398.1
**EVOO5**	0.24	0.21	4.6	4.8	1.62	1.80	0.12	0.21	0.003	0.001	357.6	544.9	434.2	569.7
**EVOO6**	0.27	0.28	8.3	6.7	1.80	1.77	0.12	0.15	0.004	0.003	308.7	266.9	563.5	561.5
**EVOO7**	0.22	0.23	5.6	5.3	1.73	1.68	0.15	0.14	0.003	0.002	340.9	341.6	572.9	420.3
**EVOO8**	0.21	0.24	5.1	5.7	1.77	1.72	0.14	0.13	0.005	0.002	336.1	306.1	509.4	439.0
**EVOO9**	0.20	0.17	4.8	4.8	1.71	1.65	0.15	0.15	0.005	0.002	459.7	437.9	569.7	456.8
**EVOO10**	0.23	0.17	5.5	6.3	1.74	1.66	0.14	0.13	0.004	0.002	263.0	282.7	517.2	338.7
**EVOO11**	0.23	0.20	4.4	5.6	1.67	1.75	0.13	0.15	0.004	0.002	467.6	300.9	423.1	432.3
**EVOO12**	0.20	0.14	4.2	7.6	1.58	1.78	0.12	0.16	0.003	0.002	369.3	310.8	450.7	393.8
**Minimum**	0.20	0.14	4.2	4.8	1.58	1.65	0.12	0.13	0.003	0.001	263.0	266.9	423.1	338.8
**Maximum**	0.27	0.28	8.3	9.6	1.80	1.80	0.15	0.21	0.005	0.003	467.6	544.9	663.6	569.7
**Mean**	**0.23**	**0.20**	**5.5**	**6.3**	**1.70**	**1.72**	**0.13**	**0.15**	**0.004**	**0.002**	**369.3**	**345.6**	**540.5**	**447.0**

**Table 2:** Content of volatile compounds in the EVOOs produced by the 12 producers across the two years.

Sample	Total VOCs (mg/kg)		Total LOX ^(A)^ (mg/kg)		(*E*)-2-Hexenal ^(B)^ (mg/kg)		Hexanal (mg/kg)		Total Rancid (mg/kg)		Total Microbial (mg/kg)		2 + 3-Methylbutanal (mg/kg)	
	2023	2024	2023	2024	2023	2024	2023	2024	2023	2024	2023	2024	2023	2024
**EVOO1**	68.5	59.0	51.4 (75%)	55.0 (93%)	45.3 (88%)	49.8 (91%)	0.473	1.034	0.392	0.065	0.077	0.507	0.042	0.040
**EVOO2**	72.8	75.9	54.6 (75%)	67.6 (89%)	47.4 (87%)	60.3 (89%)	0.433	1.087	0.221	0.080	0.041	0.543	0.030	0.046
**EVOO3**	54.6	71.2	38.6 (71%)	65.1 (91%)	33.3 (86%)	57.9 (89%)	0.623	1.237	0.601	0.073	0.075	0.500	0.057	0.050
**EVOO4**	65.3	79.2	42.2 (65%)	70.4 (89%)	36.3 (86%)	62.5 (89%)	0.309	1.031	0.269	0.070	0.064	1.600	0.048	0.043
**EVOO5**	72.1	51.0	52.4 (73%)	43.1 (85%)	46.0 (88%)	38.6 (90%)	0.690	0.557	0.614	0.060	0.048	0.178	0.034	0.054
**EVOO6**	51.2	73.2	31.3 (61%)	64.0 (87%)	26.7 (85%)	56.5 (88%)	0.339	0.706	0.601	0.074	0.066	0.275	0.041	0.064
**EVOO7**	59.2	64.1	48.8 (82%)	58.1 (91%)	43.9 (90%)	53.1 (91%)	0.535	0.861	0.538	0.060	0.122	1.610	0.066	0.037
**EVOO8**	88.8	47.5	67.2 (76%)	43.4 (91%)	59.6 (89%)	38.7 (89%)	0.424	0.781	0.339	0.088	0.119	0.554	0.054	0.033
**EVOO9**	48.7	54.6	28.4 (58%)	46.5 (85%)	23.7 (83%)	41.7 (90%)	0.467	0.560	0.506	0.043	0.078	0.234	0.055	0.035
**EVOO10**	na	72.8	na	58.5 (80%)	na	53.2 (91%)	na	0.973	na	0.127	na	0.825	na	0.026
**EVOO11**	66.3	59.9	49.6 (75%)	53.6 (89%)	44.1 (89%)	47.7 (89%)	0.274	0.742	0.415	0.066	0.096	0.643	0.056	0.044
**EVOO12**	72.1	68.8	48.9 (68%)	63.7 (93%)	44.1 (%90)	57.6 (90%)	0.307	0.983	0.521	0.072	0.090	0.134	0.042	0.040
**Minimum**	48.7	47.5	28.4 (58%)	43.1 (80%)	23.7 (83%)	38.6 (88%)	0.274	0.557	0.221	0.043	0.041	0.134	0.030	0.026
**Maximum**	88.8	79.2	67.2 (82%)	70.4 (93%)	59.6 (90%)	62.5 (91%)	0.690	1.237	0.614	0.127	0.122	1.610	0.066	0.064
**Mean**	65.4	64.8	46.7 (71%)	57.4 (89%)	40.9 (87%)	51.5 (90%)	0.443	0.879	0.456	0.073	0.080	0.634	0.048	0.043

**A**, the value in the brackets is given by the percentage value of total LOX-related VOC content of the total VOC content. **B**, the value in the brackets is given by the percentage value of total (*E*)-2-hexenal on the total LOX-related VOC content. **na**, not available. The values of EVOO 10 for 2023 are not available due to an analytical/instrumental issue.

**Table 3:** Main sensory attributes of the EVOOs produced across the two production years. The column “defect” summarizes all the defects evaluated with the IOC official profile sheet (i.e., fusty/muddy sediment, musty/humid/earth, winey/vinegary/acid/sour, frostbitten olives, rancid).

Sample	Defects		Fruitiness		Bitterness		Pungency	
	2023	2024	2023	2024	2023	2024	2023	2024
**EVOO1**	-	-	4.0	5.8	3.8	6.6	4.8	6.8
**EVOO2**	-	-	4.1	4.4	2.9	5.2	4.9	5.7
**EVOO3**	-	-	4.4	4.7	4.2	5.4	5.3	6.2
**EVOO4**	-	-	4.3	4.3	3.7	3.7	4.6	4.6
**EVOO5**	-	-	4.9	5.5	3.5	5.6	4.8	6.4
**EVOO6**	-	-	4.0	3.7	3.4	4.1	3.7	4.6
**EVOO7**	-	-	5.1	4.2	4.5	3.5	5.7	4.8
**EVOO8**	-	-	5.1	4.1	4.2	4.3	5.2	5.3
**EVOO9**	-	-	4.3	3.8	4.0	3.0	5.0	4.1
**EVOO10**	-	-	4.5	4.9	4.7	5.2	5.5	5.6
**EVOO11**	-	-	4.0	3.7	2.6	4.0	3.8	3.5
**EVOO12**	-	-	4.1	4.2	3.3	4.4	4.0	5.0

**Table 4:** Nutritional Value Score (*NVS*) of the EVOOs produced across the two production years.

NVS	EVOO 1	EVOO 2	EVOO 3	EVOO 4	EVOO 5	EVOO 6	EVOO 7	EVOO 8	EVOO 9	EVOO 10	EVOO 11	EVOO 12
**2024**	88.3	89.1	88.0	87.9	75.0	86.0	90.1	90.4	93.3	86.5	89.0	85.0
**2023**	88.5	87.0	85.0	88.6	81.9	83.1	85.0	83.1	87.8	86.9	80.4	84.5

**Table 5:** Cognitive salience index (CSI) by semantic categories.

Semantic Category	CSI	Frequency	Included Terms
**Taste and Flavor**	0.5366	527	flavor, taste, delicate, intense, body, good, full-bodied,
			tasty, sweet, aromatic, spicy, bitter, light, strong, etc.
**Aroma and Scent**	0.1795	198	scent, fragrant, smell, aroma
**Visual Appearance**	0.1739	194	color, green, yellow, transparency, dark
**Naturalness and Authenticity**	0.1344	131	natural, organic, genuine, authentic
**Health and Wellness**	0.1316	110	healthy, wholesome
**Origin and Italian Identity**	0.0978	89	Italian, local, craft
**Price and Value**	0.0503	73	price, expensive, good value

**Table 6:** Significance of the effects of individual factors and their interactions on the chemical parameters: free acidity, peroxide value, and spectrophotometric indices, as evaluated by 3-way ANOVA.

Factors	Free Acidity		Peroxide Value		K_232_		K_270_		ΔK	
	Pr(>F)	Signif.	Pr(>F)	Signif.	Pr(>F)	Signif.	Pr(>F)	Signif.	Pr(>F)	Signif.
EVOO	0.00023	***	0.1781		4.4 × 10^−8^	***	7.8 × 10^−13^	***	0.00032	***
Time	1.6 × 10^−10^	***	1.1 × 10^−14^	***	<2 × 10^−16^	***	<2 × 10^−16^	***	0.00176	**
Bottle Size	0.39749		0.0284	*	0.0887	.	0.0019	**	0.42187	
EVOO:Time	0.04919	*	0.5517		0.0363	*	1.1 × 10^−6^	***	0.07281	.
EVOO:Bottle Size	0.42427		0.5834		0.6073		0.7764		0.31631	
Time:Bottle Size	0.48375		0.0168	*	0.018	*	0.0166	*	0.35210	
EVOO:Time:Bottle Size	0.25930		0.4774		0.2631		0.7524		0.27538	

Pr(>F) indicates the level of probability that variable has no effect on that parameter, on a scale from 0 (it has an effect at 100%) to 1 (it has an effect at 0%). Significance of the effects: ‘***’, from 99.9 to 100%; ‘**’, from 99 to 99.9%; ‘*’, from 95 to 99%; ‘.’ from 90 to 99%; no symbols, from 0 to 90%.

**Table 7:** Significance of the effects of individual factors and their interactions on the total phenolic content, the main single phenols, and the hydrolysis ratio over time, as evaluated by 3-way ANOVA.

Factors	Hydroxytyrosol		Tyrosol		Oleacein		Oleocanthal		Total Phenols		Hydr. Ratio	
	Pr(>F)	Signif	Pr(>F)	Signif	Pr(>F)	Signif	Pr(>F)	Signif	Pr(>F)	Signif	Pr(>F)	Signif
EVOO	4.9 × 10^−13^	***	<2 × 10^−16^	***	<2 × 10^−16^	***	0.3538		1.6 × 10^−13^	***	0.7122	
Time	<2 × 10^−16^	***	2.2 × 10^−10^	***	<2 × 10^−16^	***	1.1 × 10^−12^	***	2.2 × 10^−7^	***	<2 × 10^−16^	***
Bottle Size	0.1720		0.6270		0.3620		0.0536	.	0.5101		0.3228	
EVOO:Time	8.3 × 10^−9^	***	7.7 × 10^−7^	***	0.6928		0.0131	*	0.1329		1.2 × 10^−7^	***
EVOO:Bottle Size	0.4440		0.8550		0.0658	.	0.9486		0.1084		0.2427	
Time:Bottle Size	0.1950		0.9280		0.0008	***	0.0054	**	0.0009	***	0.0384	*
EVOO:Time:Bottle Size	0.1780		0.1100		0.5195		0.2748		0.3131		0.1700	

Pr(>F) indicates the level of probability that variable has no effect on that parameter, on a scale from 0 (it has an effect at 100%) to 1 (it has an effect at 0%). Significance of the effects: ‘***’, from 99.9 to 100%; ‘**’, from 99 to 99.9%; ‘*’, from 95 to 99%; ‘.’ from 90 to 99%; no symbols, from 0 to 90%.

**Table 8:** Significance of the effects of individual factors and their interactions on the total tocopherol content (TTC), and on each tocopherol over time, as evaluated by 3-way ANOVA.

Factors	α-Tocopherol		β + γ-Tocopherol		δ-Tocopherol		Total Tocopherol	
	Pr(>F)	Signif.	Pr(>F)	Signif.	Pr(>F)	Signif.	Pr(>F)	Signif.
EVOO	2.47 × 10^−7^	***	<2 × 10^−16^	***	0.00768	**	0.0491	*
Time	<2 × 10^−16^	***	9.41 × 10^−16^	***	1.39 × 10^−11^	***	<2 × 10^−16^	***
Bottle Size	0.40076		0.0437	*	0.27455		0.7204	
EVOO:Time	4.72 × 10^−9^	***	2.05 × 10^−6^	***	2.74 × 10^−6^	***	8.16 × 10^−8^	***
EVOO:Bottle Size	0.00065	***	0.1288		0.21125		0.0052	**
Time:Bottle Size	0.36106		0.0767	.	0.5705		0.2958	
EVOO:Time:Bottle Size	0.0188	*	0.0279	*	0.29124		0.0251	*

Pr(>F) indicates the level of probability that variable has no effect on that parameter, on a scale from 0 (it has an effect at 100%) to 1 (it has an effect at 0%). Significance of the effects: ‘***’, from 99.9 to 100%; ‘**’, from 99 to 99.9%; ‘*’, from 95 to 99%; ‘.’ from 90 to 99%; no symbols, from 0 to 90%.

**Table 9:** Significance of the effects of individual factors and their interactions on the volatile compounds over time, as evaluated by 3-way ANOVA.

Factors	Hexanal		(*E*)-2-Hexenal		Total LOX		Rancid VOCs		Microbial VOCs		2 + 3-Methylbut	
	Pr(>F)	Signif.	Pr(>F)	Signif.	Pr(>F)	Signif.	Pr(>F)	Signif.	Pr(>F)	Signif.	Pr(>F)	Signif.
EVOO	0.0015	**	0.0131	*	0.0011	**	5.1 × 10^−7^	***	0.0648	.	2.1 × 10^−11^	***
Time	<2 × 10^−16^	***	<2 × 10^−16^	***	9.4 × 10^−15^	***	1.8 × 10^−13^	***	3.7 × 10^−8^	***	8.0 × 10^−11^	***
Bottle Size	0.0600	.	0.8801		0.8036		0.5654		0.2098		0.0504	.
EVOO:Time	0.0000	***	0.8045		0.4148		0.0062	**	0.0016	**	1.4 × 10^−7^	***
EVOO:Bottle Size	0.0030	**	0.5303		0.2308		0.0010	**	0.1292		0.1560	
Time:Bottle Size	0.3446		0.9972		0.8170		0.7117		0.0602	.	0.0152	*
EVOO:Time:Bottle Size	0.0048	**	0.6320		0.9002		0.0041	**	0.2711		8.4 × 10^−5^	***

Pr(>F) indicates the level of probability that variable has no effect on that parameter, on a scale from 0 (it has an effect at 100%) to 1 (it has an effect at 0%). Significance of the effects: ‘***’, from 99.9 to 100%; ‘**’, from 99 to 99.9%; ‘*’, from 95 to 99%; ‘.’ from 90 to 99%; no symbols, from 0 to 90%.

**Table 10:** Intensity of the main positive and negative sensory attributes in the EVOO samples during storage. n.f., not found.

	Fusty/Muddy Sediment	Winey/ Winegary	Rancid	Fruityness	Bitterness	Pungency
B t0	n.f.	n.f.	n.f.	4.0	3.9	4.7
B t1 250	n.f.	n.f.	n.f.	4.0	5.4	6.2
B t2 250	n.f.	n.f.	n.f.	3.2	6.2	4.5
B t3 250	n.f.	n.f.	n.f.	4.8	6.0	5.4
B t0	n.f.	n.f.	n.f.	4.0	3.9	4.7
B t1 500	n.f.	n.f.	n.f.	4.5	6.2	5.9
B t2 500	n.f.	n.f.	n.f.	4.3	5.8	5.4
B t3 500	n.f.	n.f.	n.f.	4.5	6.3	4.6
P t0	n.f.	n.f.	n.f.	3.8	2.9	5.3
P t1 250	n.f.	n.f.	n.f.	5.9	5.5	7.0
P t2 250	n.f.	n.f.	n.f.	5.7	5.6	6.1
P t3 250	n.f.	n.f.	n.f.	4.3	4.3	3.8
P t0	n.f.	n.f.	n.f.	3.8	2.9	5.3
P t1 500	n.f.	n.f.	n.f.	6.5	7.4	7.4
P t2 500	n.f.	n.f.	n.f.	5.0	6.4	5.5
P t3 500	n.f.	n.f.	n.f.	4.8	6.1	5.8

## Data Availability

The data presented in this study are available in the article.

## Supplementary Materials

The following supporting information can be downloaded at https://www.mdpi.com/article/10.3390/molecules30132811/s1, Table S1: Content of the main phenolic compounds and hydrolysis ratio in the EVOOs produced by the 12 producers across the two years. Table S2: Content of each tocopherol in the EVOOs produced by the 12 producers across the two years. Table S3: Percentage content of (A) each LOX-related VOC and (B) of groups of LOX-related VOCs on the total LOX-related VOC content. Table S4. Evolution over time of chemical data relating to free acidity, peroxide value, and spectrophotometric indices of the two oils (E1 = EVOO1; E2 = EVOO2) stored in two different-sized bottles. Table S5. Evolution over time of chemical data relating to phenolic compounds of the two oils (E1 = EVOO1; E2 = EVOO2) stored in two different-sized bottles. Table S6. Evolution over time of chemical data relating to tocopherols of the two oils (E1 = EVOO1; E2 = EVOO2) stored in two different-sized bottles. Table S7. Evolution over time of chemical data relating to volatile compounds of the two oils (E1 = EVOO1; E2 = EVOO2) stored in two different-sized bottles.
