# Supplementary material for: Chemical, Sensory, and Nutraceutical Profiling, and Shelf-Life Assessment of High-Quality Extra Virgin Olive Oil Produced in a Local Area near Florence (Italy)
Source: Molecules. 2025 Jun 30;30(13):2811. doi: 10.3390/molecules30132811 (PMC12251353; doi:10.3390/molecules30132811)
Supplement: Supplementary file 1 [file molecules-30-02811-s001.zip › molecules-3705102-supplementary.pdf]

Supplementary material of the manuscript

Chemical, Sensory and Nutraceutical Profiling, and Shelf-Life Assessment of High-Quality Extra Virgin Olive Oil Produced in a local area near Florence (Italy): Results from the "MontEspertOlio" project

Carlotta Breschi[1], Lorenzo Cecchi[1,*], Federico Mattagli[1], Bruno Zanoni[1], Tommaso Ugolini[1], Francesca Ieri[3], Luca Calamai[1], Maria Bellumori[2], Nadia Mulinacci[2], Fabio Boncinelli[1], Valentina Canuti[1] and Silvio Menghini[1]

[1] Department of Agricultural, Food, Environmental, and Forestry Sciences and Technologies (DAGRI) – University of Florence, via Donizetti, 6 – 50144 Firenze (Italy); C.B., carlotta.breschi@unifi.it; L.Ce., lo.cecchi@unifi.it; F.M., federico.mattagli@unifi.it; B.Z., bruno.zanoni@unifi.it; T.U., tommaso.ugolini@unifi.it; L.Ca., luca.calamai@unifi.it; F.B., Fabio.boncinelli@unifi.it; V.C., valentina.canuti@unifi.it; S.M., silvio.menghini@unifi.it

[2] Department of NEUROFARBA, University of Florence, Via Ugo Schiff 6, 50019 Sesto F.no, Florence, Italy; N.M., nadia.mulinacci@unifi.it; M.B., maria.bellumori@unifi.it.

[3] National Research Council of Italy (CNR), Institute of Bioscience and BioResources (IBBR), Sesto Fiorentino (Florence), Italy; francesca.ieri@cnr.it

* Correspondence: lo.cecchi@unifi.it; Tel.: +39 055 0552755517

Table S1. Content of the main phenolic compounds and hydrolysis ratio in the EVOOs produced by the 12 producers across the two years.

Sample	Hydroxytyrosol (mg/kg)		Tyrosol (mg/kg)		Oleacein (mg/kg)		Oleocantal (mg/kg)		Lignans (mg/kg)		Hydrolysis ratio (mg/kg)	
	**2023**	**2024**	**2023**	**2024**	**2023**	**2024**	**2023**	**2024**	**2023**	**2024**	**2023**	**2024**
**EVOO1**	3.6	6.9	3.5	5.4	222.3 (39.6%)	121.8 (29.7%)	139.7 (24.9%)	123.2 (30.1%)	45.6	18.2	1.1	2.3
**EVOO2**	3.6	1.0	5.9	2.7	177.4 (29.8%)	152.5 (38.5%)	174.6 (29.3%)	122.0 (30.8%)	46.6	15.3	1.7	0.9
**EVOO3**	2.9	0.7	5.3	2.0	207.6 (31.3%)	117.2 (29.4%)	160.4 (24.2%)	129.5 (32.5%)	52.1	18.2	1.4	0.7
**EVOO4**	3.2	1.4	4.8	2.3	152.6 (35.1%)	137.3 (24.1%)	192.3 (44.3%)	122.0 (21.4%)	57.9	19.7	1.2	0.9
**EVOO5**	2.6	1.7	4.4	2.9	117.6 (20.9%)	175.3 (31.2%)	138.2 (24.5%)	144.8 (25.8%)	36.2	38.5	1.6	0.8
**EVOO6**	2.2	1.4	4.5	2.3	231.3 (40.4%)	186.2 (44.3%)	147.6 (25.8%)	178.8 (42.5%)	34.8	21.5	1.2	0.6
**EVOO7**	2.7	0.9	5.3	2.2	104.9 (20.6%)	182.5 (41.6%)	163.6 (32.1%)	105.3 (24.0%)	64.2	20.2	1.4	0.7
**EVOO8**	5.7	0.9	3.8	3.1	121.0 (21.2%)	143.9 (31.5%)	161.8 (28.4%)	141.7 (31.0%)	36.9	21.7	1.9	0.9
**EVOO9**	3.1	1.2	6.0	3.1	183.8 (35.5%)	191.6 (56.6%)	144.0 (27.8%)	115.6 (34.1%)	45.1	20.7	1.6	0.9
**EVOO10**	3.2	0.6	3.9	2.0	86.8 (20.5%)	92.7 (21.4%)	175.6 (41.5%)	117.0 (27.1%)	55.2	24.1	1.4	0.8
**EVOO11**	3.0	1.4	1.8	2.4	73.9 (16.4%)	116.4 (29.6%)	113.0 (25.1%)	123.6 (31.4%)	44.4	29.1	1.1	0.9
**EVOO12**	2.6	0.7	2.4	2.0	139.9 (33.1%)	118.0 (34.8%)	143.1 (33.8%)	128.3 (37.9%)	35.7	18.8	1.1	0.7
**Minimum**	2.2	0.6	1.8	2.0	73.9 (16.4%)	92.7 (21.4%)	113.0 (25.1%)	105.3 (24.0%)	34.8	15.4	1.1	0.6
**Maximum**	5.7	6.9	6.0	5.4	231.3 (40.4%)	191.6 (56.6%)	192.3 (44.3%)	178.8 (42.5%)	64.2	38.5	1.9	2.3
**Mean**	3.2	1.6	4.3	2.7	151.6	144.6	154.5	129.3	46.2	22.2	1.4	0.9

Table S2. Content of each tocopherols in the EVOOs produced by the 12 producers across the two years.

Sample	α (mg/kg)		β+γ (mg/kg)		δ (mg/kg)	
	2023	2024	2023	2024	2023	2024
**EVOO 1**	319.2	247.9	64.6	19.9	3.9	1
**EVOO 2**	355.5	283.9	49.8	29.4	2.5	3.8
**EVOO 3**	310.2	291.6	60.9	33.6	1.9	1.6
**EVOO 4**	315.1	294.0	51.9	28.5	2.3	nd
**EVOO 5**	305.0	498.1	49.3	46.8	3.3	nd
**EVOO 6**	265.6	247.3	40.3	19.6	2.8	nd
**EVOO 7**	321.9	307.4	19.0	34.3	nd	nd
**EVOO 8**	315.3	281.4	20.7	24.6	nd	nd
**EVOO 9**	392.1	388.6	64.4	45.8	3.2	3.5
**EVOO 10**	232.3	253.5	29.2	27.6	1.5	1.5
**EVOO 11**	396.5	258.9	66.5	42.0	4.5	nd
**EVOO 12**	321.6	280.7	44.9	27.1	2.9	3
**Minimum**	232.3	247.3	19.0	19.6	1.5	1.0
**Maximum**	396.5	498.1	66.5	46.8	4.5	3.8
**Mean**	320.9	302.8	46.8	31.6	2.9	2.4

Table S3. Percentage content of A) each LOX-related VOC and B) of groups of LOX-related VOCs on the total LOX-related VOCs content

A)

Sample	1-Penten-3-one		Hexanal		(E)-2-Pentenal		(Z)-3-Hexenal		(E)-2-Hexenal		(E,E)-2,4-Hexadienal		1-Penten-3-ol		·2-Penten-1-ol	
	2023	2024	2023	2024	2023	2024	2023	2024	2023	2024	2023	2024	2023	2024	2023	2024
EVOO 1	1.87	2.29	0.91	1.87	0.16	0.14	0.89	0.77	87.51	90.40	0.58	0.16	0.80	0.91	0.14	0.11
EVOO 2	2.63	2.64	0.79	1.60	0.21	0.15	0.77	0.68	86.35	89.05	0.48	0.11	0.83	0.86	0.16	0.09
EVOO 3	3.02	1.97	1.60	1.89	0.24	0.10	0.85	0.63	85.81	88.84	0.61	0.11	1.09	0.76	0.20	0.08
EVOO 4	3.09	2.33	0.72	1.46	0.25	0.11	0.82	0.59	85.22	88.72	0.76	0.11	1.03	0.90	0.24	0.09
EVOO 5	2.04	2.53	1.31	1.29	0.20	0.10	0.79	0.59	87.31	89.42	0.50	0.13	0.80	1.35	0.13	0.12
EVOO 6	2.87	3.10	1.08	1.10	0.27	0.15	0.76	0.60	84.84	88.23	0.54	0.11	1.05	1.10	0.22	0.12
EVOO 7	1.89	2.24	1.09	1.48	0.16	0.12	0.87	0.60	89.32	91.28	0.60	0.10	0.64	0.79	0.12	0.09
EVOO 8	2.08	2.61	0.63	1.79	0.17	0.16	0.90	0.73	88.07	88.96	0.73	0.25	0.46	1.06	0.13	0.14
EVOO 9	3.54	2.92	1.63	1.20	0.24	0.13	0.68	0.54	83.12	89.67	0.66	0.13	1.62	1.05	0.25	0.13
EVOO 10	-	1.51	-	1.66	-	0.10	-	0.70	-	90.80	-	0.22	-	0.60	-	0.10
EVOO 11	2.75	2.34	0.55	1.38	0.22	0.12	0.76	0.59	88.23	88.81	0.62	0.14	0.42	0.78	0.15	0.08
EVOO 12	2.29	2.22	0.62	1.54	0.20	0.12	0.69	0.66	89.64	90.29	0.51	0.12	0.39	0.72	0.13	0.08
Minimum	1.87	1.51	0.55	1.10	0.16	0.10	0.68	0.54	83.12	88.23	0.48	0.10	0.39	0.60	0.12	0.08
Maximum	3.54	3.10	1.63	1.89	0.27	0.16	0.90	0.77	89.64	91.28	0.76	0.25	1.62	1.35	0.25	0.14
Mean	2.55	2.39	0.99	1.52	0.21	0.13	0.80	0.64	86.86	89.54	0.60	0.14	0.83	0.91	0.17	0.10

Sample	(Z)-2-Penten-1-ol		1-Hexanol		-3-Hexen-1-ol		(E)-2-Hexen-1-ol		Hexyl acetate		(Z)-3-Hexenyl acetate		(E)-2-Hexenyl acetate	
	2023	2024	2023	2024	2023	2024	2023	2024	2023	2024	2023	2024	2023	2024
EVOO 1	1.07	0.70	1.01	0.57	3.30	1.01	1.16	0.67	0.05	0.10	0.55	0.28	nd	0.01
EVOO 2	1.22	0.73	0.92	0.87	3.00	1.19	1.78	1.82	0.07	0.03	0.80	0.17	nd	0.00
EVOO 3	1.42	0.64	0.69	1.22	2.31	1.13	1.35	2.26	0.08	0.05	0.73	0.32	nd	0.01
EVOO 4	1.60	0.70	1.06	1.08	2.35	1.69	1.49	1.70	0.21	0.05	1.15	0.46	nd	0.01
EVOO 5	1.03	0.90	0.84	0.78	1.88	0.89	2.83	1.60	0.06	0.03	0.28	0.26	nd	0.00
EVOO 6	1.42	0.90	1.16	0.75	1.87	1.66	3.45	1.49	0.10	0.08	0.37	0.60	nd	0.01
EVOO 7	0.89	0.68	0.90	0.57	1.75	1.25	1.61	0.49	0.04	0.03	0.13	0.26	nd	0.00
EVOO 8	0.98	0.85	1.42	0.86	1.88	1.52	2.17	0.89	0.08	0.03	0.32	0.13	nd	0.00
EVOO 9	1.73	0.83	1.27	0.76	2.73	0.98	1.41	1.25	0.14	0.05	0.98	0.34	nd	0.00
EVOO 10	-	0.57	-	0.95	-	1.08	-	1.33	-	0.09	-	0.29	-	0.01
EVOO 11	1.13	0.66	1.35	0.93	1.36	1.59	2.24	1.05	0.05	0.16	0.16	1.36	nd	0.01
EVOO 12	0.82	0.61	1.01	0.88	1.28	1.16	2.21	1.05	0.05	0.13	0.17	0.42	nd	0.00
Minimum	0.82	0.57	0.69	0.57	1.28	0.89	1.16	0.49	0.04	0.03	0.13	0.13	nd	0.00
Maximum	1.73	0.90	1.42	1.22	3.30	1.69	3.45	2.26	0.21	0.16	1.15	1.36	nd	0.01
Mean	1.21	0.73	1.06	0.85	2.16	1.26	1.97	1.30	0.08	0.07	0.51	0.41	nd	0.01

B)

Sample	Ketones (%)		Other aldehydes (%)		Alcohols (%)		Esters (%)	
	2023	2024	2023	2024	2023	2024	2023	2024
**EVOO1**	1.9	2.3	2.0	2.8	5.9	2.9	0.6	0.4
**EVOO2**	2.6	2.6	1.8	2.4	5.8	3.2	0.9	0.2
**EVOO3**	3.0	2.0	2.7	2.6	5.1	3.5	0.8	0.4
**EVOO4**	3.1	2.3	1.8	2.2	5.9	4.2	1.4	0.5
**EVOO5**	2.1	2.5	2.3	2.0	4.0	3.4	0.3	0.3
**EVOO6**	2.9	3.1	2.1	1.9	4.7	4.3	0.5	0.7
**EVOO7**	1.9	2.2	2.1	2.2	3.6	3.0	0.2	0.3
**EVOO8**	2.1	2.6	1.7	2.7	4.3	3.7	0.4	0.2
**EVOO9**	3.6	2.9	2.6	1.9	6.9	3.3	1.1	0.4
**EVOO10**	-	1.5	-	2.5	-	3.0	-	0.4
**EVOO11**	2.8	2.3	1.5	2.1	3.5	4.8	0.2	1.5
**EVOO12**	2.3	2.2	1.5	2.3	3.0	3.3	0.2	0.6
**Minimum**	1.9	1.5	1.5	1.9	3.0	2.9	0.2	0.2
**Maximum**	3.6	3.1	2.7	2.8	6.9	4.8	1.4	1.5
**Mean**	2.6	2.4	2.0	2.3	4.8	3.6	0.6	0.5

Table S4. Evolution over time of chemical data relating to free acidity, peroxide value, and spectrophotometric indices of the two oils (E1 = EVOO1; E2 = EVOO2) stored in two different-size bottles.

	Free acidity	Peroxide value	K_{232}	K_{270}	ΔK
B t0	0.22 ± 0.01	4.5 ± 0.1	1.65 ± 0.02	0.13 ± 0.01	0.003 ± 0.001
B250 t1	0.26 ± 0.01	5.5 ± 0.1	1.86 ± 0.02	0.14 ± 0.01	0.004 ± 0.001
B250 t2	0.32 ± 0.02	8.6 ± 0.2	2.06 ± 0.05	0.15 ± 0.01	0.005 ± 0.001
B250 t3	0.31 ± 0.05	9.8 ± 1.3	2.25 ± 0.09	0.17 ± 0.01	0.004 ± 0.001
B500 t1	0.25 ± 0.02	6.0 ± 0.2	1.85 ± 0.01	0.14 ± 0.01	0.005 ± 0.001
B500 t2	0.35 ± 0.04	8.1 ± 0.1	2.04 ± 0.06	0.15 ± 0.00	0.003 ± 0.002
B500 t3	0.28 ± 0.03	7.3 ± 0.6	2.19 ± 0.04	0.18 ± 0.01	0.004 ± 0.001
P t0	0.21 ± 0.01	5.1 ± 0.2	1.62 ± 0.01	0.13 ± 0.01	0.003 ± 0.001
P250 t1	0.22 ± 0.03	5.9 ± 0.1	1.70 ± 0.01	0.12 ± 0.01	0.004 ± 0.001
P250 t2	0.29 ± 0.02	8.4 ± 0.1	1.91 ± 0.01	0.13 ± 0.01	0.004 ± 0.001
P250 t3	0.28 ± 0.03	9.7 ± 1.7	2.16 ± 0.15	0.15 ± 0.00	0.003 ± 0.001
P500 t1	0.19 ± 0.02	5.8 ± 0.2	1.74 ± 0.01	0.13 ± 0.01	0.004 ± 0.001
P500 t2	0.27 ± 0.04	8.1 ± 0.2	1.91 ± 0.02	0.14 ± 0.01	0.004 ± 0.001
P500 t3	0.29 ± 0.02	8.6 ± 2.1	1.98 ± 0.07	0.15 ± 0.00	0.003 ± 0.001

Table S5. Evolution over time of chemical data relating to phenolic compounds of the two oils (E1 = EVOO1; E2 = EVOO2) stored in two different-size bottles.

	Hydroxytyrosol	Tyrosol	Oleacein	Oleocanthal	TPC	Hydrolysis ratio
B t0	3.6	3.5	177.9	67.9	618.8	1.14%
B250 t1	4.8	4.2	147.4	66.9	569.9	1.59%
B250 t2	5.5	3.6	178.5	77.3	608.0	1.50%
B250 t3	13.5	4.8	144.5	62.2	579.9	3.17%
B500 t1	5.7	4.3	144.9	64.6	561.6	1.78%
B500 t2	5.5	3.2	166.1	70.0	536.9	1.60%
B500 t3	13.2	4.9	153.2	65.5	603.9	3.01%
P t0	3.6	5.9	128.8	68.0	560.9	1.68%
P250 t1	3.0	6.8	98.6	66.3	500.1	1.95%
P250 t2	2.5	4.7	128.6	80.0	513.2	1.41%
P250 t3	6.4	5.6	94.0	62.8	472.4	2.54%
P500 t1	3.1	6.4	98.9	60.6	492.9	1.92%
P500 t2	2.9	5.1	127.9	77.7	499.0	1.59%
P500 t3	6.6	5.5	112.6	64.8	517.4	2.34%

Table S6. Evolution over time of chemical data relating to tocopherols of the two oils (E1 = EVOO1; E2 = EVOO2) stored in two different-size bottles.

	TOCOPHEROLS			
	δ	β+γ	α	Totale
B t0	3.9 ± 0.1	64.6 ± 1.5	319.2 ± 4.1	387.7 ± 5.6
B250 t1	2.2 ± 0.1	61.9 ± 1.1	300.8 ± 4.3	364.9 ± 5.4
B250 t2	1.7 ± 0.2	56.9 ± 1.0	306.2 ± 4.4	366.8 ± 5.6
B250 t3	1.6 ± 0.5	61.0 ± 0.1	281.0 ± 6.4	345.5 ± 6.8
B500 t1	2.2 ± 0.1	64.8 ± 1.8	296.4 ± 2.8	363.4 ± 4.4
B500 t2	2.5 ± 1.0	59.6 ± 0.5	301.8 ± 5.2	365.8 ± 5.2
B500 t3	1.7 ± 0.3	59.8 ± 0.6	258.6 ± 3.7	322.0 ± 3.9
P t0	2.5 ± 0.1	49.8 ± 1.1	355.5 ± 4.9	407.8 ± 4.5
P250 t1	2.4 ± 0.1	46.5 ± 1.7	294.3 ± 5.4	343.2 ± 4.9
P250 t2	2.3 ± 0.6	42.5 ± 1.5	299.7 ± 2.8	346.5 ± 3.4
P250 t3	1.5 ± 0.4	48.8 ± 1.6	276.2 ± 6.4	328.5 ± 8.0
P500 t1	2.5 ± 0.1	46.5 ± 0.6	299.7 ± 5.1	348.8 ± 4.9
P500 t2	2.3 ± 0.1	42.5 ± 1.1	305.2 ± 9.9	351.9 ± 10.8
P500 t3	1.4 ± 0.4	49.3 ± 1.2	285.1 ± 8.6	337.8 ± 10.1

Table S7. Evolution over time of chemical data relating to volatile compounds of the two oils (E1 = EVOO1; E2 = EVOO2) stored in two different-size bottles.

Mean (mg/kg)	Total LOX	(E)-2-Hexenal	Hexanal	Rancid VOCs	2+3-methylbutanal	Microbial VOCs
B t0	51.217	45.317	0.473	0.392	0.042	0.077
B t1 250	50.555	44.561	0.617	0.453	0.041	0.077
B t2 250	32.021	25.913	1.130	0.424	0.058	0.545
B t3 250	35.785	25.938	3.219	0.658	0.050	0.349
B t0	51.217	45.317	0.473	0.392	0.042	0.077
B t1 500	48.654	41.862	0.580	0.416	0.033	0.044
B t2 500	29.849	24.859	0.900	0.385	0.057	0.709
B t3 500	35.798	26.612	2.575	0.629	0.047	0.263
P t0	54.454	47.445	0.433	0.221	0.030	0.041
P t1 250	49.893	43.164	0.625	0.284	0.034	0.045
P t2 250	37.170	28.268	1.146	0.278	0.041	0.289
P t3 250	37.930	30.651	2.053	0.501	0.044	0.614
P t0	54.454	47.445	0.433	0.221	0.030	0.041
P t1 500	52.070	45.883	0.607	0.247	0.045	0.078
P t2 500	37.450	28.296	1.166	0.286	0.040	0.211
P t3 500	41.630	29.794	2.274	0.559	0.032	0.193
